# Novel Norovirus GII.4 Variant, Shanghai, China, 2012

**DOI:** 10.3201/eid1908.130026

**Published:** 2013-08

**Authors:** Zhen Shen, Fangxing Qian, Yang Li, Yunwen Hu, Zhenghong Yuan, Jun Zhang

**Affiliations:** Shanghai Public Health Clinical Center, Shanghai, China (Z. Shen, Y. Hu, J. Zhang);; Changning District Center Hospital, Shanghai (F. Qian);; Shanghai Dongfang Hospital, Shanghai (Y. Li);; Fudan University, Shanghai (Z. Yuan)

**Keywords:** norovirus, norovirus GII.4 variant, viruses, epochal evolution, epidemics, Shanghai, China, enteric infections

**To the Editor:** Norovirus (NoV) has been identified as one of the major causal agents of nonbacterial, acute gastroenteritis in humans (*1*). The genetic diversity among NoVs is great, and human strains have been classified into 3 genogroups (GI, GII, and GIV). Despite this diversity, in recent years only a few strains, primarily those of genogroup II, genotype 4 (GII.4), have been responsible for most cases and outbreaks worldwide (*1**,**2*).

The pattern of epochal evolution of NoV is ongoing, and novel GII.4 variants emerge, which replace previously dominant strains and cause new pandemics. Surveillance systems worldwide showed an increase in NoV activity in late 2012 (*3*). Molecular data shared through NoroNet (www.rivm.nl/en/Topics/Topics/N/NoroNet) suggest that this increase is related to the emergence of a new GII.4 variant, termed Sydney_2012 (*3*). We found that this novel GII.4 variant also emerged in Shanghai, China, and caused increased levels of NoV activity during October–December 2012.

During July 2011–December 2012, fecal specimens from 748 outpatients (≥16 years of age) with acute gastroenteritis who visited 1 of the 2 sentinel hospitals in Shanghai were collected and stored at Shanghai Public Health Clinical Center at −70°C. Molecular detection of GI and GII NoV was performed by using conventional reverse transcription PCR as described (*4*). Full-length viral protein 1 and 639 bp of the 3′ RNA-dependent RNA polymerase gene of 4 randomly selected GII-positive strains were amplified (*5**–**7*). NoV genotypes were classified on the basis of a 280-bp region for GI and a 305-bp region for GII by using the Automated Genotyping Tool (www.rivm.nl/mpf/norovirus/typingtool).

A total of 77 patients showed positive results for GII NoV. An increase in GII NoV activity was observed during October–December in 2012; the detection rate was 46.08% (47 cases in 102 outpatients). The prevalence of GII NoV during the same period in 2011 was low; the detection rate was 6.90% (8 cases in 116 outpatients). Genotyping analysis of the strains detected in these 3 months in 2012 (39 strains were sequenced) showed that except for 1 GII.6 strain and 3 GII.4 2006b strains, the other 35 strains sequenced all belong to the new established cluster of GII.4, termed Sydney_2012. Retrospective analysis indicated that the novel GII.4 variant had already been detected in 2 outpatients during September 2011 in Shanghai.

Phylogenetic analysis of full-length capsid nucleotide sequences for 4 strains randomly selected from the new cluster indicated a novel GII.4 pattern, and new strains clustering separately from previously identified GII.4 pandemic strains (Figure). On the basis of BLAST (http://blast.ncbi.nlm.nih.gov/Blast.cgi) searches, the most closely related NoVs (98%–100% nucleotide identity) were 4 GII.4 viruses recently detected in Australia and Hong Kong. The new GII.4 strains detected in Shanghai also clustered with these strains, a finding that was supported by bootstrap values >70% (Figure). The 3′ end of RNA-dependent RNA polymerase gene sequences also confirmed that the new GII.4 strains were recombinants, with a GII.e polymerase and GII.4 capsid (*3*).

**Figure:**
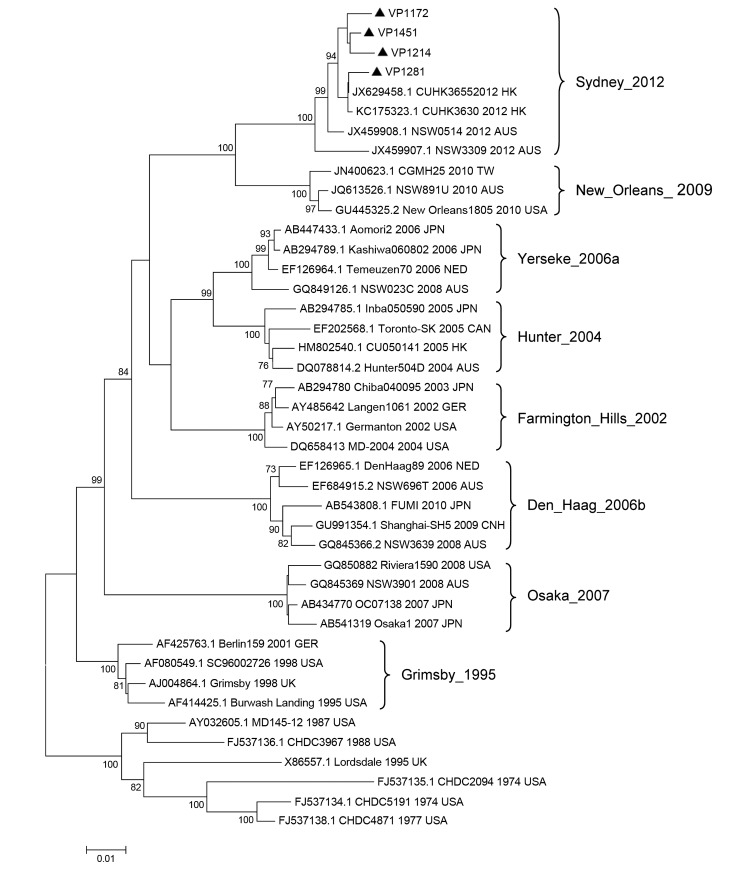
Phylogenetic tree of norovirus GII.4 capsid nucleotide sequences, Shanghai, China. The dendrogram was constructed by using the neighbor-joining method in MEGA version 5.0 (*8*). Bootstrap resampling (1,000 replications) was used, and bootstrap values ≥70% are shown. Black triangles indicate the 4 representative strains detected in Shanghai (GenBank accession nos. KC456070–KC456073). Reference sequences were obtained from GenBank and are indicated by GenBank accession number, strain name, year, and country of detection. Locations and years on the right indicate previously dominant GII.4 variants. HK, Hong Kong; AUS, Australia; TW, Taiwan; USA, United States; JPN, Japan; NED, the Netherlands; CAN, Canada; GER, Germany; CHN, China; UK, United Kingdom. Scale bar indicates distances between sequence pairs.

Despite improved control measures to combat NOV, this highly infectious agent continues to cause a large number of epidemics of gastroenteritis globally (approximately every 2 years), and most epidemics have been associated with emergence of a novel GII.4 cluster (*9*). The new cluster reported in the present study was first detected in Australia in March, 2012, followed by detection in France, New Zealand, Japan, the United Kingdom, the United States, and Hong Kong, where increased levels of NoV activity in late 2012 compared with previous seasons were also observed (*3*). This novel GII.4 strain has also emerged in Shanghai, China, and caused increased levels of sporadic cases during October–and December 2012. This new variant has common ancestors, dominant NoV GII.4 variants Osaka_2007 and New Orleans_2009, but is phylogenetically distinct from them. Amino acid changes are present in major epitopes located in the P2 domain, a finding that is consistent with observations from previous epidemics (*3*).
